# Bowel Ischemia From Suspected Non-occlusive Mesenteric Ischemia: A Case Report

**DOI:** 10.7759/cureus.83175

**Published:** 2025-04-29

**Authors:** Mohammad Anzal Rehman, Ramna Abdulrahman, Bayan Awad, Amna Almukhayet, Rishi Prasad

**Affiliations:** 1 Emergency Department, Mediclinic City Hospital, Dubai, ARE; 2 Emergency Department, Mohammed Bin Rashid University of Medicine and Health Sciences, Dubai, ARE

**Keywords:** abdominal pain, dihydropyrimidine dehydrogenase (dpd) deficiency, emergency department, non-occlusive mesenteric ischemia, pain out of proportion

## Abstract

Acute ischemia of the bowel is a rare but life-threatening diagnosis in the emergency department (ED). While early detection is key to appropriate management, the utility of investigative modalities is limited by poor sensitivity and specificity. This report outlines the case of a 37-year-old female patient who presented to a tertiary hospital ED with severe, generalized, abdominal pain for several hours after eating at a restaurant. She had a known history of treatment for breast cancer, on chemotherapy, and was also diagnosed with dihydropyrimidine dehydrogenase (DPD) deficiency. Laboratory investigations, including C-reactive protein and full blood counts (FBCs), were not significantly suggestive of bowel ischemia, and CT abdomen and pelvis with intravenous (IV) contrast and angiogram revealed no obvious vascular occlusion. Severe, persistent pain out of proportion to physical examination raised suspicion of a non-occlusive mesenteric ischemia (NOMI), and a diagnostic laparotomy was performed, uncovering an infarcted duodenal segment that underwent resection and anastomosis. Post-op, the patient recovered well and was discharged without complications. Given the absence of typical risk factors, such as advanced age, atrial fibrillation, coagulopathy, cardiac disease, or a low-flow state in our patient, we explore the association of chemotherapeutic agents with NOMI, as well as the implications of DPD deficiency. A high index of suspicion for mesenteric ischemia should be maintained for any patient with severe, intractable abdominal pain, out of proportion to the clinical examination, even in the absence of conventional risk factors. Patients with malignancies may require additional consideration of ischemia risk from the use of chemotherapeutic agents, with possibly an increased risk with platinum-based compounds. Known DPD deficiency may also worsen chemotherapeutic drug-related toxicities, although its implication in ischemia is not well established.

## Introduction

Acute ischemia of the bowel is associated with mortality rates of up to 80% [1]. While several investigative modalities have been proposed to detect the presence of bowel ischemia, their utility is limited by poor sensitivity and specificity. Most acute bowel ischemia occurs as a result of embolism or thrombosis of a major vessel, typically the superior mesenteric artery (SMA). Where no vascular occlusion is present, the mechanism is termed as non-occlusive mesenteric ischemia (NOMI), accounting for an infrequent 20% of cases, and usually encountered in older, critically ill patients with persistent low-flow states, such as cardiac failure and shock [2].

The mechanism of NOMI is less understood compared to its occlusive counterpart, where a large vessel occlusion can often be more readily identified. Most cases of NOMI occur in patients with reduced cardiac output or where multi-organ hypoperfusion has occurred. Patients tend to be critically unwell, with circulatory supply-demand mismatches, like in septic shock, and this may be a confounder in the relatively higher mortality rates of over 70% in patients with NOMI [3]. Unfortunately, the absence of a definitive, localized occlusion also leads to a more complicated, delayed, and often extensive surgical resection as non-viable, necrotic lesions of bowel secondary to NOMI tend to be diffuse and patchy [2].

While the presence of cancer may induce prothrombotic states that have been linked to both thrombosis and embolism in patients [4], its association with NOMI has not been well established. However, there are several reports in cancer patients that seem to indicate a possible relation to chemotherapy use with the development of symptoms of NOMI.

This report outlines the case of a 37-year-old female with seemingly atypical risk factors for thromboembolism or ischemia presenting with acute, persistent, severe abdominal pain. In the absence of remarkable diagnostic results, clinical suspicion alone was pivotal in guiding appropriate management. The possible role of chemotherapy agents in the development of NOMI in this case is then explored.

## Case presentation

A 37-year-old female presented to a tertiary hospital emergency department (ED) with severe, generalized abdominal pain after eating food from an outside restaurant earlier that day. The patient was diagnosed with adenocarcinoma of colon few months ago and underwent left hemicolectomy for an obstructing tumor, following which she was commenced on adjuvant chemotherapy, i.e., FOLFOX regimen (5-fluorouracil + oxaliplatin). The chemotherapy was started several weeks back, but the patient was being monitored closely for features of mucositis due to a recently discovered dihydropyrimidine dehydrogenase (DPD) genotype. This genotype increased risk of mucositis due to a DPD deficiency, so the patient was undergoing routine testing for liver function tests (LFTs) every two days, lastly done the day before the ED encounter, with no significant abnormalities noted.

**Figure 1:**
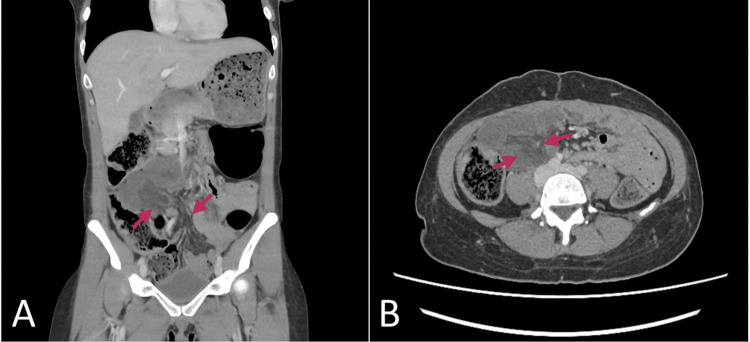
CT abdomen and pelvis with IV contrast, with (A) coronal and (B) axial views demonstrating minimal bowel wall edema and lack of contrast enhancement in the duodenum's region (arrows)

Upon assessment, the vital signs revealed tachycardia 110 beats per minute, with an elevated respiratory rate 22 breaths per minute and a blood pressure of 135/98 mmHg. Physical examination was significant for vague tenderness in the right upper and left lower quadrants. A venous blood gas showed no significant acid-base imbalance, with a lactate value of 1.2. The abdominal pain did not abate despite administration of parenteral acetaminophen, ketorolac, esomeprazole, high-dose fentanyl, and morphine, as well as sub-dissociative doses of ketamine in the ED. As part of the work-up for severe abdominal pain, a CT abdomen and pelvis with intravenous (IV) contrast was performed at one-hour post arrival to the ED, revealing minimal bowel edema in the duodenum with a lack of contrast infiltration near the region (Figure 1). 

Laboratory investigations revealed no leukocytosis and neutrophilia, with a C-reactive protein (CRP) <1 mg/L (Table 1).

**Table 1:** Laboratory results in the initial emergency department (ED) encounter eGFR: estimated glomerular filtration rate, CRP: C-reactive protein, LFT: liver function test, HCT (PCV): hematocrit (packed cell volume), RDW: red cell distribution width, MPV: mean platelet volume, MCH: mean corpuscular hemoglobin, MCHC: mean corpuscular hemoglobin concentration, MCV: mean corpuscular volume

Test item	Reference range	Units	Value
Creatinine	53 – 97	Umol/L	74.4
eGFR	> 60	mL/min/1.73m^2^	91
CRP	0.0 – 5.0	Mg/L	<0.6
Lipase	13.0 – 60.0	U/L	32.3
Urea nitrogen	2.14 – 7.14	Mmol/L	7.41
LFT			
Total bilirubin	<21.0	Umol/L	3.75
Direct biliruibin	<=7.0	umol/L	2.05
Alkaline phosphatase (ALP)	35-104	U/L	50.9
ALT (alanine amino transferase)	<35	U/L	14.6
Albumin (ALB)	35.00 – 52.00	g/L	38
AST (aspartate aminotransferase)			16.7
Protein (total)	64-83	g/L	71.7
FBC (full blood count)			
HCT (PCV)	31-42	%	33.3
RDW	12.3-17.7	%	17.7
MPV	7.9-10.8	fL	9.61
Basophils %		%	0.22
Basophils	0-0.1	10^3 ^/uL	0.01
Eosinophils %		%	3.30
Eosinophils	0-0.5	10^3 ^/uL	0.21
Hemoglobin	11.5-16.0	g/dL	10.9
Lymphocytes %		%	62.61
Lymphocytes	1.10-3.10	10^3 ^/uL	4.06
MCH	24.7-32.8	Pg	24.7
MCHC	32.3-35.6	g/dL	32.7
MCV	80.0-100.0	fL	75.5
Monocytes %		%	5.98
Monocytes	0.2-0.9	10^3 ^/uL	0.39
Neutrophils %		%	27.89
Neutrophils	1.90-8.20	10^3 ^/uL	1.81
Platelet count	150-450	10^3 ^/uL	298
Red cell count	3.80-5.20	10^6 ^/uL	4.41
White cell count	4-11	10^3 ^/uL	6.49

**Figure 2:**
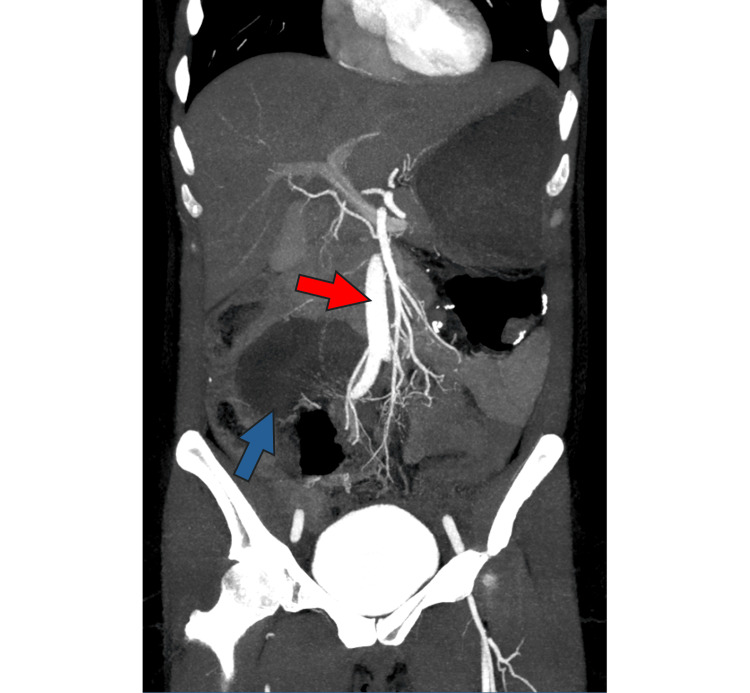
CT angiography of the abdomen and pelvis. As reported, the findings included the patient's abdominal aorta (red arrow) and its visceral branches, with no sign of arterial occlusion. Patent portal vein, mesenteric veins, inferior vena cava, and their major tributaries, no sign of thrombosis-occlusion. Some decreased enhancement in the walls of the distal ileal small bowel and mesenteric edema in the right lower quadrant noted (blue arrow), raising concern for small bowel ischemia.

A repeat CT angiogram was performed approximately five hours after the ED arrival, with no arterial or venous occlusion reported (Figure 2).

**Figure 3:**
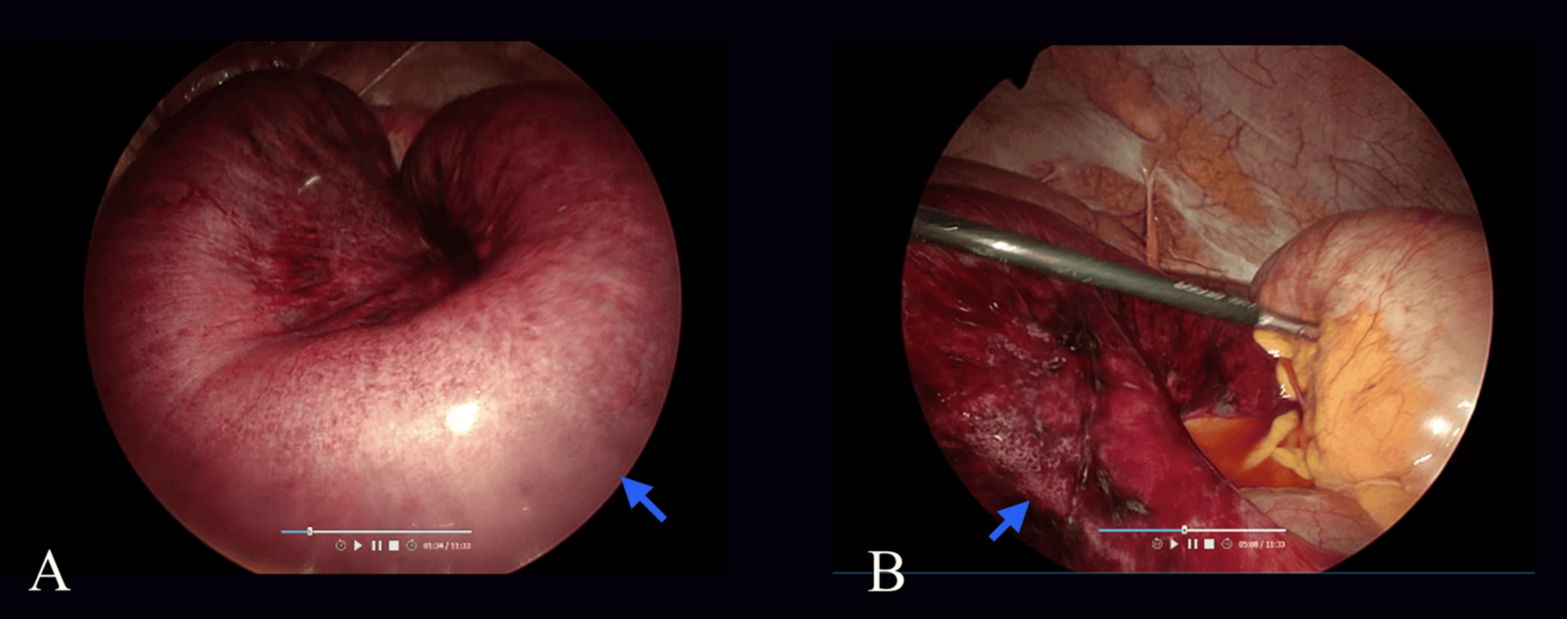
Explorative laparotomy intraoperative images. Ileal small bowel and mesenteric edema with segments of infarction (arrows).

Serial lactate levels one hour apart and CRP levels four hours apart remained unelevated. General Surgery was consulted, and the patient was admitted for serial abdominal examinations and pain management, with the suspicion of an underlying mesenteric ischemia despite the negative vascular findings on CT imaging. Approximately eight hours after initial ED presentation, given the continued, severe abdominal pain, a diagnostic laparoscopy was performed, which revealed an infarcted segment of duodenum (Figure 3) with patent mesenteric arteries. Hence, a diagnosis of NOMI was made for the patient.

The laparoscopy was therefore advanced to a mini laparotomy, and a segmental resection of the necrotic segments of small bowel and an end-to-end anastomosis were performed. Histopathology of the resected bowel showed features consistent with bowel ischemia, with lamina propria hemorrhage, superficial epithelial necrosis, serosal inflammation, and no evidence of malignancy. There was good recovery postoperatively, and the patient eventually discharged pain-free with good functional outcomes.

## Discussion

Acute mesenteric ischemia (AMI) is a rare but potentially fatal condition, accounting for approximately 0.09-0.2% of ED visits, with mortality rates of more than 50% in confirmed cases. Early detection allows for expeditious management, usually with surgical resection of infarcted bowel [5]. AMI may be classed as either occlusive or non-occlusive, with the most common type attributable to SMA embolism, although occurrence secondary to thrombosis has been increasing in frequency. Typical risk factors of embolic disease include advanced age, atrial fibrillation, and coagulopathy [6].

NOMI is a rare sub-type of AMI, with risk increased around persistent low-flow states, such as cardiac disease, shock, and hypovolemia, which could be exacerbated by the actions of vasoactive agents, such as in patients who are critically ill within the intensive care unit. Characteristic signs of mesenteric ischemia include nausea, vomiting, diarrhea, and rectal bleeding in around 15-45% of patients. However, the predominant complaint, especially in NOMI, is abdominal pain, present in around 95% of patients with mesenteric ischemia [7].

Despite a myriad of proposed lab markers and diagnostic modalities, very few have shown to be reliable at identifying mesenteric ischemia. A systematic review in 2013 by Cudnik et al. looked at various serum markers, such as lactate, D-dimer, fatty acid-binding protein (FABP), and alpha-glutathione S-transferase (GST), but found no reliable evidence supporting the use for any of these in diagnosing mesenteric ischemia. D-dimer had the highest sensitivity but low specificity. Lactate is moderately sensitive but also non-specific test, which may have a role in serial monitoring. Other findings that may be present include leukocytosis, elevated hematocrit, amylase, troponin, and metabolic acidosis [8].

The diagnosis of mesenteric ischemia relies on a high index of suspicion in a patient with abdominal pain, especially one that is out of proportion, with an otherwise unremarkable physical examination. The presence of laboratory values such as metabolic acidosis with elevated lactate, elevated D-dimer, or leukocytosis should give rise to suspicion for AMI, prompting early computed tomography angiography (CTA) to more definitively assess for bowel ischemia [5].

Our case follows a patient that is unique in that she does not possess any of the risk factors typically associated with AMI. Notably, her age was below 45 years, and she had no history of cardiac disease or atrial fibrillation and no known history of thromboembolism. We evaluated for a potential association of mesenteric ischemia with the use of chemotherapeutic agents, specifically 5- fluorouracil and oxaliplatin, especially in the setting of the patient’s DPD deficiency.

Malignancy and its associated treatment in the form of chemotherapy both confer in affected patients an increased thromboembolic risk. Occlusion of blood vessels may be a result of either vascular toxicity or drug-related endothelial damage that results in a prothrombotic state, more commonly encountered with the use of platinum-based chemotherapeutic agents (e.g., cisplatin, carboplatin) [9]. The presence of occlusive mesenteric ischemia secondary to chemotherapy use is a rare occurrence, with only 16 reported cases found in published literature, usually with taxane-based agents (e.g., paclitaxel, docetaxel, and cabazitaxel) [10].

Arterial thromboembolism in platinum-based agents is more characteristically encountered with cisplatin, with at least six cases identified in the literature, two of which involved mesenteric ischemia [11]. Thromboembolic risk has not been as commonly reported with 5-fluorouracil use. However, increases in its toxicity have been well established in the setting of DPD deficiency [12].

While the patient in our case did undergo chemotherapy with a platinum-based compound (oxaliplatin) and 5-fluorouracil, acute bowel ischemia occurred in the absence of an identifiable large vessel occlusion; hence, a diagnosis of NOMI was made.

Several mechanisms have been proposed for the development of NOMI in patients. Splanchnic blood flow decreases during meals and even with moderate exercise. Periods of whole-body stress, such as in hypovolemic or hemorrhagic shock, tend to shunt blood flow away from the gastrointestinal organs preferentially to other vital end-organs. Therefore, low-flow states may reduce oxygen delivery to intestinal tissue. However, abnormal oxygen-carrying capacity of the blood, impaired cellular uptake, or abnormal oxygen utilization are all mechanisms by which the supply-demand mismatch may produce NOMI.

Risk factors for the development of NOMI include an age more than 50 years, low cardiac output states (e.g., congestive heart failure), end-stage renal disease requiring hemodialysis, liver failure, and reduced splanchnic blood flow from vasoactive drugs [13].

In the absence of advanced age, recent sepsis, heart failure, or other conventional risk factors, the possibility of chemotherapy-induced NOMI, with or without the influence of DPD deficiency was explored. A literature search over the past 20 years using the MEDLINE and PMC (PubMed Central) database linking NOMI with either chemotherapy or DPD deficiency yielded 19 results. Articles in languages other than English were excluded from the search. Only four studies were selected for relevance, comprising a total of six cases of NOMI associated with any chemotherapy use. One case was a diagnosis on autopsy and hence was excluded [14]. Another case outlined NOMI in a 68-year-old male patient with NOMI seven months after the use of cisplatin and etoposide use for small cell lung cancer with splenic metastasis, but where the incidence of NOMI was ultimately ruled to be caused by septicemia rather than chemotherapy use [15]. The main features of the remaining four cases are highlighted in Table 2 [16,17]. Notably, no significant results were found to establish an association of mesenteric ischemia with DPD deficiency.

**Table 2:** Summary of published cases of non-occlusive mesenteric ischemia (NOMI) associated with chemotherapy use

Article	Published	Case	Patient age/gender	Cancer site	Chemotherapy	Onset of symptoms post chemotherapy	Surgical findings
Nagano H et al.	2020	1	74/Male	Oropharyngeal	Oral S-1/5-fluorouracil	5 days	Necrosis of the small intestinal, ascending colon, and transverse colon
		2	68/Male	Hypopharyngeal	Docetaxel, cisplatin and fluorouracil (TPF)	7 days	No surgery due to clinical instability
		3	82/Male	Hypopharyngeal	N/A (only radiotherapy)	3 months after radiotherapy	Small intestine necrosis
Matsuzawa M et al.	2015	4	74/Female	Nodular melanoma with lung metastasis	Carboplatin and paclitaxel	Unspecified	Necrosis of terminal ileum and the ascending and transverse colon

The exact mechanism of NOMI secondary to chemotherapy may not easily be determined due to the lack of data surrounding this phenomenon. While chemotherapy agents have been implicated in thromboembolic incidents, there is no vascular occlusion in NOMI. Direct mucosal irritation may lead to direct intestinal toxicity, pneumatosis, perforation, bleeding, and transmural ischemia, particularly with cisplatin and irinotecan. Drug-related endothelial damage and coagulation cascade hyper-activation are both forms of vascular toxicity that, when involving bowel vessels, may indirectly lead to prothrombotic states and ischemia. This is most commonly encountered with drugs such as cisplatin and gemcitabine [9].

Ultimately, while there was clear evidence of bowel ischemia in our case, the definitive cause for the occurrence of NOMI in our patient is difficult to establish. Management therefore centered around resection of visualized ischemic bowel, with optimized circulatory support and care to avoid any further reductions to splanchnic blood flow. The role of prophylactic anticoagulation is currently understudied for vascular occlusions or ischemia secondary to chemotherapy [11].

## Conclusions

AMI is a rare, but important diagnosis to consider in patients with severe, intractable abdominal pain out of proportion to physical examination. Where conventional laboratory markers or diagnostic imaging such as CT angiogram for abdomen and pelvis do not reveal signs of vascular occlusion, a diagnosis of NOMI should strongly be considered. The use of chemotherapy agents may represent an important risk factor in the development of NOMI, with taxane- and platinum-based agents more commonly implicated in prothrombotic events. Further research is warranted into the exact mechanisms and risks associated with chemotherapeutic agents for the development of mesenteric ischemia.
